# Annotations

**Published:** 1888-09-01

**Authors:** 


### ANNOTATIONS.

**Ambulances Still Wanted.**

OUR annotation on this subject last week has resulted in some interesting correspondence in the daily papers. Now that Parliament is not sitting, and that "big gooseberries" have all been relegated to the jam-pot, our enterprising daily contemporaries cannot do better than open their correspondence and leader columns to some of the more pressing hospital questions. Here and there an ambulance may be found connected with a general hospital. What may be the antiquity or efficiency of the structure cannot always be ascertained. At St. Mary's, we are informed on the best authority, there is a good and useful machine. Perhaps, however, in too many other places there is either no ambulance at all or one which would fall to pieces if an attempt were made to use it. The following true story probably well illustrates the case of most of those hospitals which possess what they call "an ambulance": A few years ago a fire broke out in the public hall of a small Yorkshire town. The building was of considerable magnitude, four or five storeys high. "Run for the fire engine!" was everybody's cry. No sooner said than done. Scores of willing feet hastened to the hiding-place of the fire engine under the belfry of the parish church. Delusive hope! The moment strong hands were laid on the extinguisher it incontinently went to pieces. The next parish, a couple of miles off, was tried, with a like result. A market-town, eight miles away, was sought on a galloping horse, and its engine was also found *hors de combat*. At length, about three hours from the commencement of the outbreak, an engine was brought, which speedily put out the fire; and all the more speedily because the building was by that time reduced to bare walls and ashes. The truth about the ambulance question seems to be that whilst the towns and districts needing this kind of aid are numbered by the hundred, ambulance carriages which are worth the name may be counted on the fingers. London, which should be the very centre and pioneer of all hospital progress, seems to be hopelessly behind in this department of first aid. Edinburgh, according to a correspondent in the *Daily News*, appears to be as conspicuously in advance. The question is urgent, and it is more than probable that The Hospitals Association will bring it prominently before the public within the next few months. In the meantime the daily papers may render good service by leaving Mrs. Mona Caird severely alone, and guiding the energies of their contributors and correspondents into this and other comparatively untrodden regions of hospital life and management.

**The Money Value of a Live Man.**

WE are coming to express everything in terms of pounds, shillings, and pence. According to Mr. Edwin Chadwick, president for the present year of the Association of Public Sanitary Inspectors, the actual average money value of a living Briton, of whatever age, is £159. We must confess that this average strikes us as rather high, though we would not for a moment enter into competition either with Dr. Farr or Mr. Chadwick on such a subject. The President of the Association of Sanitary Inspectors has an object in thus calling attention to the money value of human beings. That object is to get questions affecting the public health considered in the right quarter. He is of opinion, as many others of us have been, that public authorities, so far from considering human beings as of any great state value, have looked upon many of them rather in the light of public nuisances. Many men who write M.P. after their names, consider wars and pestilences as public benefactors, because they clear out the surplus population. Even some who have tender and gentle hearts have been inclined to think that the economists of the destructive class had some reason on their side. Mr. Chadwick, who is well entitled to speak, and Dr. Farr, whose authority on the subject is higher than that of all the members of the House of Commons put together, agree in an emphatic protest against this barbarous and unscientific position. Life, like time, is money. The waste of life is the waste of money. Bad sanitation is the waste of life. In London alone, says Mr. Chadwick, there are more than 35,000 lives lost annually from defective sanitation. These lives represent a value to the State of more than five and a-half millions sterling. Will this make legislators better sanitarians? We doubt it. The truth of the matter is that the business of legislation is conducted in so confused and inefficient a way in this country that legislators have not time for sanitary questions. Devolution and decentralisation are imperative if work is to be done. The State, as an entity, can take no account of sanitary details. It is the people themselves who suffer from the lives that are annually thrown away; and the people, who live in crowded towns, and who themselves are constantly running the risk of unnecessarily throwing away their own lives, must insist upon better sanitation. Until individual men and women have the intelligence to understand and the energy to act upon what the doctors teach them, lives will continue to be wasted and homes to be wrecked as wantonly as before. As yet, few men seem to see that State responsibility is their responsibility. What the State does or leaves undone is done or left undone by each individual voter. If the State is foolish or criminal the ultimate blame must rest upon voters alone.

**Poor John Aldcroft!**

IT must be admitted, though surely with sorrow, that the philosophers who regard the ultimate principle of human action as a "struggle for survival" have good grounds for their conclusion. Everywhere, in all ranks, among those of the highest education as among those of the lowest, there is a constant tendency to revert to first principles; to carry out "the good old rule, the simple plan; that he may take who has the power, and he may keep who can." Poor John Aldcroft, a lad of nineteen, as all the papers have proclaimed, fell from the shafts of his loaded waggon while fast asleep, and was run over and killed. Aldcroft, a Cheshire lad, had been working almost night and day for at least a week—how much longer it is not stated—and although in charge of horses and a loaded waggon he found it quite impossible to keep awake. No one knows better than the medical man the terrible effects of want of sleep. The poor lad Aldcroft, kept awake merely to serve his employer's greed, was at an age when endurance beyond a certain point is impossible. So little sleep as he had had would probably have driven a more highly nervous man to madness or to suicide. Aldcroft being an out-door labourer, and probably of an inactive brain, simply kept awake as long as Nature would permit him, and then, in spite of everything, went to sleep. How profound and deathlike that slumber was the event proved; he fell from his seat under the wheels of his waggon and paid for it with his life. If those who have since written to the newspapers speak truth—as they certainly do—for the present writer in the course of his medical night duties in a London suburb, has himself seen hundreds of loaded waggons at all hours of the early morning, wending their slow way along with sleepy horses and sleepier men—if they speak truth, is there no call for these poor, helpless men to be protected by the laws of a civilised country? No man can work both night and day. We cannot use up human beings as we use up cattle—killing them slowly or quickly at our convenience. What does our civilisation amount to if it cannot protect the helpless against such hopeless slavery as this? The world consists of the strong and the weak. Is it really true that civilisation and Christianity have brought us no further than this, that if a man be too weak to defend himself, he shall have no defence at all? In this country churches and chapels are numbered by tens of thousands, and in them all there is professedly taught the religion of one who proclaimed as his most elementary doctrine that all men are brothers, whilst God is their common father. If this be our religion, we are either so intellectually stupid that we cannot understand even its elements, or so thoroughly hypocritical that though we outwardly conform, inwardly we do not believe it. Every man who has a vote must hold himself responsible for the death of John Aldcroft, or, if not for his death, at least for the slavery under which thousands of Aldcroft's fellows still groan, and for all future deaths which may come in the same way. Nothing is more shocking and horrible than a perfect religion on the tongue, and cynicism greed, and murder in the heart.

